# Prevalence of Depression and Anxiety Among Qassim University Students During the COVID-19 Pandemic

**DOI:** 10.7759/cureus.34866

**Published:** 2023-02-11

**Authors:** Mansour I Alsoghair, Abdulmajeed S Alharbi, Abdullah I Aldekhail, Yousef O Alharbi, Feras A Alkhuzayyim, Abdullah F Alowais, Ziyad I Almohaimeed

**Affiliations:** 1 Community Medicine, Qassim University, Buraydah, SAU; 2 College of Medicine, Qassim University, Buraydah, SAU

**Keywords:** covid-19, qassim university, mental health, depression, coronavirus disease 2019, anxiety

## Abstract

Introduction

Depression and anxiety are two types of mental disorders. Individuals with depression usually experience depressed mood, loss of interest or enjoyment, and reduced energy, leading to increased fatigability that diminishes their activity. Meanwhile, anxiety disorders refer to a group of mental disorders characterized by feelings of anxiety and fear. The coronavirus disease 2019 (COVID-19) pandemic has led to mass quarantine, isolation, and lockdowns worldwide, which have impacted the population's mental health. In Saudi Arabia, a study showed that 17.1% and 10.5% of the population had moderate-to-severe features of depression and anxiety, respectively. Demonstrating the prevalence of depression and anxiety in educational institutions is essential. This study aimed to measure the prevalence of depression and anxiety among students at Qassim University during the COVID-19 pandemic.

Methods

This cross-sectional study was conducted at Qassim University, Saudi Arabia. The students were selected using a multistage random sampling technique. An online questionnaire was sent to the selected students via e-mail and social media platforms. The questionnaire contained three parts: the first part included socio-demographic questions, the second part contained the Patient Health Questionnaire-9 (PHQ-9) to measure depression, and the third part contained the Generalized Anxiety Disorder-7 (GAD-7) questionnaire to measure anxiety.

Results

In total, 411 university students completed the questionnaire (response rate = 75%). The prevalence of depression and anxiety was 40.6% and 29.4%, respectively. Females had higher levels of depression and anxiety than men (p < 0.001). The College of Arabic Language and Social Studies (CALSS) had the highest prevalence of depression and anxiety (42.9% and 30.6%, respectively).

Conclusion

We found a high post-pandemic prevalence of depression and anxiety among the students at Qassim University. Our findings demonstrate the need for psychological intervention programs for the students of Qassim University.

## Introduction

Depression and anxiety disorders are two types of mental disorders. Individuals with depression usually experience depressed mood, loss of interest or enjoyment, and reduced energy, leading to increased fatigability that diminishes their activity. Depression has many other symptoms that can be more serious, like having suicidal thoughts and performing acts of self-harm [1,2]. The classification of depression is mainly based on the severity, duration, and course of symptoms [3,4]. Meanwhile, anxiety disorders refer to a group of mental disorders characterized by feelings of anxiety and fear [2]. Generalized anxiety disorder (GAD) is an anxiety disorder in which anxiety is generalized, persistent, and unrelated to particular environmental circumstances. Patients with GAD complain of nervousness, muscular tension, sweating, palpitations, dizziness, and other anxiety-related symptoms. GAD is often associated with chronic environmental stress [1].

Coronavirus disease 2019 (COVID-19) was declared a global pandemic by the World Health Organization (WHO) on March 11, 2020 [5]. The COVID-19 pandemic has led to mass quarantine, isolation, and lockdowns worldwide, impacting the mental health of the global population [6]. The COVID-19 pandemic has increased the prevalence of stress, post-traumatic stress disorder, anxiety, and depression. It also increased the incidence of suicide and worsened the condition of obsessive-compulsive disorder patients [7]. COVID-19 can also cause neurological complications such as olfactory dysfunction, headaches, and cognitive impairments [8]. Interestingly, mental health may act as a factor in determining the outcome of COVID-19 because mental health may increase the risk of vaccine hesitancy [9]. During the COVID-19 pandemic, video-conferencing applications increased as a way of communication. Unfortunately, video-conferencing exacerbated body dysmorphic disorder, which is known to be associated with depression and anxiety [10]. Before the COVID-19 pandemic, the latest estimated prevalence of depression and anxiety disorders globally was 3.44% (2-6%) and 3.8% (2.5-7%), respectively, in 2017 [11]. However, these estimates changed during the COVID-19 pandemic. A meta-analysis of community-based studies showed that the global prevalence of depressive disorders increased from 3.44% in 2017 to 25% in 2020 [12]. Another meta-analysis showed that the prevalence of depression and anxiety was 37% and 41%, respectively [13].

Regarding social media, it is essential to promote mental health awareness during pandemics. One published article reviewed the role of social media during pandemics and mentioned many advantages: "maintains connectivity among people; facilitates continuous consultation with physicians through telemedicine; reduces loneliness, anxiety, and depression." However, social media use can be detrimental to mental health. The disadvantages mentioned are as follows: "gives a sense of insecurity to oneself and leads to psychiatric illnesses; promotes advertisement of the use of illegal substances; attracts cyberbullying, which can give a negative impact on adolescents" [14].

Saudi Arabia imposed partial and complete curfews during the COVID-19 pandemic several times on different regions and cities. In May of the previous year, it even imposed a complete nationwide lockdown that included Eid al-Fitr [15-17]. These measures have affected the mental health of the general population of Saudi Arabia. Two studies in Saudi Arabia used the Depression, Anxiety, and Stress Scale-21 items (DASS-21) as a study instrument to assess the mental health status of their participants. The first study published in December 2020 showed that 17.1% and 10.5% of the general population in Saudi Arabia have moderate to severe features of depression and anxiety, respectively. The second study was conducted in February 2021 and reported that 43.2% and 34.6% of the students in Saudi Arabia had moderate to extremely severe symptoms of depression and anxiety, respectively [18,19].

Demonstrating the prevalence of depression and anxiety disorders in educational institutions is important. This would expose the size of the challenge, thus guiding the allocation of resources needed to promote public mental health. Public mental health promotion is provided through mental health awareness programs, free mental health screenings, and the implementation of other programs and activities that would help public mental health. In addition, promoting public mental health among university students helps them complete their studies successfully [20].

Qassim University is an educational institution with approximately 70,000 students. We aimed to measure the prevalence of depression and anxiety among these students during the COVID-19 pandemic and compare the prevalence among different colleges [21].

## Materials and methods

Study design and setting

This study is a cross-sectional study, and it began in November 2021 and ended in July 2022. This study was conducted at Qassim University, Saudi Arabia.

Sampling strategy

Students were selected using a two-stage random sampling technique. In the first stage of our sampling strategy, we allocated colleges at Qassim University into three strata based on similarities between their themes and areas of interest and then selected one college from each stratum using simple random sampling. At this stage, the College of Public Health and Informatics (CPHI), College of Architecture and Planning (CAP), and College of Arabic Language and Social Studies (CALSS) were selected.

In the second stage of our sampling strategy, we selected students from each college through a stratified random sampling technique. The number of students selected from each college was proportional to the total number of students enrolled.

Measurement instrument and data collection

An online questionnaire was sent to the selected students via e-mail and social media platforms. The questionnaire contained six multiple-choice questions to identify the socio-demographic characteristics of the participants (age, sex, marital status, nationality, family's monthly income, and chronic morbidity). The questionnaire also contained pre-validated Arabic versions of the Patient Health Questionnaire-9 (PHQ-9) and Generalized Anxiety Disorder-7 (GAD-7) questionnaire [22]. The PHQ-9 and GAD-7 are tools used to measure depression and anxiety, respectively.

The PHQ-9 consists of nine items and four choices, and based on the answers to these nine items, participants were assigned a score out of 27 (Table 1).

**Table 1:** The Patient Health Questionnaire-9 (PHQ-9)

	Not at all	Several days	More than half the days	Nearly every day
Little interest or pleasure in doing things	0	1	2	3
Feeling down, depressed, or hopeless	0	1	2	3
Trouble falling or staying asleep, or sleeping too much	0	1	2	3
Feeling tired or having little energy	0	1	2	3
Poor appetite or overeating	0	1	2	3
Feeling bad about yourself or that you are a failure or have let yourself or your family down	0	1	2	3
Trouble concentrating on things, such as reading the newspaper or watching television	0	1	2	3
Moving or speaking so slowly that other people could have noticed or the opposite, being so fidgety or restless that you have been moving around a lot more than usual	0	1	2	3
Thoughts that you would be better off dead or hurting yourself in some way	0	1	2	3

Based on students' total scores in PHQ-9, they were divided into five categories; scores 0-4, 5-9, 10-14, 15-19, and 20-27 were categorized into “no depression,” “mild depression,” “moderate depression,” “moderately severe depression,” and “severe depression,” respectively. The cut-off score for diagnosing depression was 10.

Meanwhile, the GAD-7 questionnaire consists of seven items and four choices; however, participants’ final score is assigned out of 21 (not 27) since GAD-7 has only seven items (Table 2).

**Table 2:** The Generalized Anxiety Disorder-7 (GAD-7) questionnaire

	Not at all	Several days	More than half the days	Nearly every day
Feeling nervous, anxious, on edge, or worrying a lot about different things	0	1	2	3
Not being able to stop or control worrying	0	1	2	3
Worrying too much about different things	0	1	2	3
Trouble relaxing	0	1	2	3
Being so restless that it is hard to sit still	0	1	2	3
Becoming easily annoyed or irritable	0	1	2	3
Feeling afraid as if something awful might happen	0	1	2	3

Based on students’ total scores in GAD-7, they were divided into four categories; scores between 0 and 4, 5 and 9, 10 and 14, and 15 and 21 were categorized into “no anxiety,” “mild anxiety,” “moderate anxiety,” and “severe anxiety,” respectively. Similar to depression, the cut-off score for diagnosing anxiety was 10.

Statistical analysis

Data analysis was performed using the RStudio software (R version 4.1.1, RStudio Team, Boston, MA). Scale reliability was assessed using Cronbach’s alpha test. Frequencies and percentages were used to express categorical data, and medians and interquartile ranges (IQRs) were used to present numerical data. The factors associated with different levels of depression and anxiety were investigated using Fisher’s exact test for count data. Statistical significance was set at p < 0.05.

## Results

In total, 411 university students completed the questionnaire (response rate = 75%). The students of the CALSS represented the largest proportion of the sample (91.2%), whereas the proportions of those studying in the CPHI and CAP were 5.4% and 3.4%, respectively. Regarding the students' socio-demographic characteristics, more than half were female (51.9%), and less than two-thirds were between 20 and 23 years of age (63.4%). The majority of the students were single (95.2%) and Saudi nationals (96.0%). Less than half of the students (49.3%) had a family monthly income of <10,000 Saudi riyals (SAR), more than one-third (35.2%) had a family monthly income between 10,000 and 20,000 SAR, and 15.5% had a monthly family income of >20,000 SAR. The majority of students (80.2%) were free from any chronic diseases (Table 3).

**Table 3:** Socio-demographic characteristics Note: Questions regarding the socio-demographic characteristics were not mandatory to answer thus not all participants’ socio-demographic characteristics were collected. CPHI: College of Public Health and Informatics; CAP: College of Architecture and Planning; CALSS: College of Arabic Language and Social Studies.

Variable	Category	N (%)
Age (years)	<20	52 (15.3%)
	20 to <23	215 (63.4%)
	23 or more	72 (21.2%)
Sex	Male	195 (48.1%)
	Female	210 (51.9%)
Marital status	Single	336 (95.2%)
	Married	17 (4.8%)
Nationality	Saudi	338 (96.0%)
	Non-Saudi	14 (4.0%)
Monthly income of the family (Saudi riyal)	<10,000	168 (49.3%)
	10,000 to 20,000	120 (35.2%)
	>20,000	53 (15.5%)
Chronic disease	None	284 (80.2%)
	Asthma	21 (5.9%)
	Diabetes	4 (1.1%)
	Hypertension	1 (0.3%)
	Heart disease	3 (0.8%)
	Others	41 (11.6%)
College	CPHI	22 (5.4%)
	CAP	14 (3.4%)
	CALSS	375 (91.2%)

Based on the PHQ-9, the median (IQR) depression score was 8.0 (4.0-12.0), and the reliability analysis showed that the scale was generally reliable (Cronbach’s alpha = 0.830). Approximately 26.0% of the participants had no depression, whereas mild, moderate, moderately severe, and severe depression levels were prevalent in 33.3%, 24.6%, 10.9, and 5.1% of the participants, respectively (Table 4).

**Table 4:** Levels of depression

Depression levels	Percentage of participants
None	26.0%
Mild	33.3%
Moderate	24.6%
Moderately severe	10.9%
Severe	5.1%

According to the cut-off score for diagnosing depression, the prevalence of depression in our sample was 40.6%.

Regarding anxiety, which was assessed using the GAD-7, the median (IQR) score was 6.0 (3.0-10.5) with a Cronbach’s alpha coefficient of 0.883. Almost one-third of the students had no anxiety (37.7%), whereas mild, moderate, and severe anxiety levels were reported in 32.8%, 16.5%, and 12.9% of participants, respectively (Table 5).

**Table 5:** Levels of anxiety

Anxiety levels	Percentage of participants
None	37.7%
Mild	32.8%
Moderate	16.5%
Severe	12.9%

According to the cut-off score for diagnosing GAD, the prevalence of GAD in our sample was 29.4%.

Regarding factors associated with depression, being female was associated with higher levels of depression. In our sample, 75% of those with “severe depression” were females, while 25% were males; for “moderately severe depression,” 68.2% were females and 31.8% were males, for “moderate depression,” 60.2% were females and 39.8% were males, for “mild depression,” 50.4% were females and 49.6% were males, and for “no depression,” 34.9% were females and 65.1% were males. According to the cut-off score, the prevalence of depression among females was 49.5%, and that among males was 29.7%. The sex differences were statistically significant (p < 0.001). Being free of chronic diseases is associated with lower levels of depression. The proportion of students with “no depression” or “mild depression” who were free of chronic diseases was 90.6% and 83.2%, respectively. In comparison, the proportions of those with “moderate depression,” “moderately severe depression,” and “severe depression” who were free of chronic diseases were 73.8%, 64.4%, and 77.8%, respectively (p = 0.013). Importantly, the proportion of students with severe anxiety increased with increasing depression severity. In particular, “severe anxiety” was prevalent among 0% of students with “no depression” and 4.4% of those with “mild depression.” However, the prevalence of “severe anxiety” was 9.9% in those with “moderate depression,” 42.2% in students with “moderately severe depression,” and 85.7% in students with “severe depression” (p < 0.001). The levels of depression did not differ significantly between the groups based on age, marital status, nationality, monthly family income, and college (Table 6).

**Table 6:** Factors associated with depression CPHI: College of Public Health and Informatics; CAP: College of Architecture and Planning; CALSS: College of Arabic Language and Social Studies.

Variable	Category	Levels of depression					
		No (N = 107)	Mild (N = 137)	Moderate (N = 101)	Moderately severe (N = 45)	Severe (N = 21)	P-value
Age	<20	17 (20.5%)	17 (14.3%)	9 (11.5%)	8 (19.5%)	1 (5.6%)	0.785
	20 to <23	49 (59.0%)	78 (65.5%)	51 (65.4%)	25 (61.0%)	12 (66.7%)	
	23 or more	17 (20.5%)	24 (20.2%)	18 (23.1%)	8 (19.5%)	5 (27.8%)	
Sex	Male	69 (65.1%)	68 (49.6%)	39 (39.8%)	14 (31.8%)	5 (25.0%)	<0.001
	Female	37 (34.9%)	69 (50.4%)	59 (60.2%)	30 (68.2%)	15 (75.0%)	
Marital status	Single	82 (95.3%)	118 (94.4%)	82 (97.6%)	39 (95.1%)	15 (88.2%)	0.465
	Married	4 (4.7%)	7 (5.6%)	2 (2.4%)	2 (4.9%)	2 (11.8%)	
Nationality	Saudi	84 (98.8%)	122 (97.6%)	79 (94.0%)	37 (90.2%)	16 (94.1%)	0.090
	Non-Saudi	1 (1.2%)	3 (2.4%)	5 (6.0%)	4 (9.8%)	1 (5.9%)	
Monthly income of the family	<10,000	38 (46.3%)	57 (47.1%)	42 (51.2%)	19 (50.0%)	12 (66.7%)	0.782
	10,000 to 20,000	29 (35.4%)	42 (34.7%)	31 (37.8%)	13 (34.2%)	5 (27.8%)	
	>20,000	15 (18.3%)	22 (18.2%)	9 (11.0%)	6 (15.8%)	1 (5.6%)	
Chronic disease	None	77 (90.6%)	104 (83.2%)	62 (73.8%)	27 (64.3%)	14 (77.8%)	0.013
	Asthma	4 (4.7%)	7 (5.6%)	6 (7.1%)	3 (7.1%)	1 (5.6%)	
	Diabetes	1 (1.2%)	1 (0.8%)	1 (1.2%)	1 (2.4%)	0 (0.0%)	
	Hypertension	0 (0.0%)	0 (0.0%)	0 (0.0%)	1 (2.4%)	0 (0.0%)	
	Heart disease	0 (0.0%)	3 (2.4%)	0 (0.0%)	0 (0.0%)	0 (0.0%)	
	Others	3 (3.5%)	10 (8.0%)	15 (17.9%)	10 (23.8%)	3 (16.7%)	
College	CPHI	7 (6.5%)	11 (8.0%)	4 (4.0%)	0 (0.0%)	0 (0.0%)	0.060
	CAP	7 (6.5%)	5 (3.6%)	0 (0.0%)	1 (2.2%)	1 (4.8%)	
	CALSS	93 (86.9%)	121 (88.3%)	97 (96.0%)	44 (97.8%)	20 (95.2%)	
Anxiety	No	91 (85.0%)	47 (34.3%)	15 (14.9%)	2 (4.4%)	0 (0.0%)	<0.001
	Mild	13 (12.1%)	63 (46.0%)	49 (48.5%)	10 (22.2%)	0 (0.0%)	
	Moderate	3 (2.8%)	21 (15.3%)	27 (26.7%)	14 (31.1%)	3 (14.3%)	
	Severe	0 (0.0%)	6 (4.4%)	10 (9.9%)	19 (42.2%)	18 (85.7%)	

Regarding factors associated with anxiety, similar to that of depression, the female sex was associated with higher anxiety levels. Considering the univariate analysis of factors associated with anxiety, the results showed that the proportion of females increased significantly with the increased severity of anxiety. Females represented 35.9% of students with “no anxiety,” 57.0% of students with “mild anxiety,” 63.6% of students with “moderate anxiety,” and 70.6% of those with “severe anxiety” (p < 0.001). According to the cut-off score, the prevalence of anxiety among females was 37.1%, and that among males was 20.0%. No other factors were associated with anxiety (Table 7).

**Table 7:** Factors associated with anxiety CPHI: College of Public Health and Informatics; CAP: College of Architecture and Planning; CALSS: College of Arabic Language and Social Studies.

Variable	Category	Levels of anxiety				
		No (N = 155)	Mild (N = 135)	Moderate (N = 68)	Severe (N = 53)	P-value
Age	<20	20 (15.7%)	18 (16.5%)	9 (15.3%)	5 (11.4%)	0.886
	20 to <23	77 (60.6%)	67 (61.5%)	39 (66.1%)	32 (72.7%)	
	23 or more	30 (23.6%)	24 (22.0%)	11 (18.6%)	7 (15.9%)	
Sex	Male	98 (64.1%)	58 (43.0%)	24 (36.4%)	15 (29.4%)	<0.001
	Female	55 (35.9%)	77 (57.0%)	42 (63.6%)	36 (70.6%)	
Marital status	Single	125 (95.4%)	113 (95.8%)	56 (94.9%)	42 (93.3%)	0.900
	Married	6 (4.6%)	5 (4.2%)	3 (5.1%)	3 (6.7%)	
Nationality	Saudi	127 (97.7%)	111 (94.1%)	56 (94.9%)	44 (97.8%)	0.444
	Non-Saudi	3 (2.3%)	7 (5.9%)	3 (5.1%)	1 (2.2%)	
Monthly income	<10,000	55 (44.0%)	63 (54.8%)	33 (57.9%)	17 (38.6%)	0.118
	10,000 to 20,000	45 (36.0%)	35 (30.4%)	20 (35.1%)	20 (45.5%)	
	>20,000	25 (20.0%)	17 (14.8%)	4 (7.0%)	7 (15.9%)	
Chronic disease	None	112 (85.5%)	92 (78.6%)	46 (76.7%)	34 (73.9%)	0.229
	Asthma	9 (6.9%)	6 (5.1%)	2 (3.3%)	4 (8.7%)	
	Diabetes	1 (0.8%)	2 (1.7%)	1 (1.7%)	0 (0.0%)	
	Hypertension	0 (0.0%)	0 (0.0%)	0 (0.0%)	1 (2.2%)	
	Heart disease	1 (0.8%)	2 (1.7%)	0 (0.0%)	0 (0.0%)	
	Others	8 (6.1%)	15 (12.8%)	11 (18.3%)	7 (15.2%)	
College	CPHI	11 (7.1%)	7 (5.2%)	3 (4.4%)	1 (1.9%)	0.405
	CAP	8 (5.2%)	4 (3.0%)	0 (0.0%)	2 (3.8%)	
	CALSS	136 (87.7%)	124 (91.9%)	65 (95.6%)	50 (94.3%)	

For the comparison between different colleges, the prevalence of depression was highest among the students of CALSS (42.9%). Meanwhile, the prevalence of depression among students of CPHI and CAP was 18.1% and 14.2%, respectively (Table 8).

**Table 8:** Depression levels by college CPHI: College of Public Health and Informatics; CAP: College of Architecture and Planning; CALSS: College of Arabic Language and Social Studies.

College	None	Mild	Moderate	Moderately severe	Severe	Total
CPHI	31.8%	50.0%	18.2%	0.0%	0.0%	100%
CAP	50.0%	35.7%	0.0%	7.14%	7.14%	100%
CALSS	24.8%	32.3%	25.9%	11.7%	5.3%	100%

The prevalence of anxiety was also the highest among the students of CALSS (30.6%), and surprisingly, the prevalence of anxiety among the students of CPHI and CAP was similar to that of depression (18.1% and 14.2%, respectively) (Table 9).

**Table 9:** Anxiety levels by college CPHI: College of Public Health and Informatics; CAP: College of Architecture and Planning; CALSS: College of Arabic Language and Social Studies.

College	None	Mild	Moderate	Severe	Total
CPHI	50.0%	31.8%	13.6%	4.54%	100%
CAP	57.1%	28.6%	0.0%	14.3%	100%
CALSS	36.3%	33.0%	17.33%	13.33%	100%

## Discussion

In this study, we assessed the prevalence of depression and anxiety among students at Qassim University during the COVID-19 pandemic using the PHQ-9 and GAD-7 questionnaires to measure depression and anxiety, respectively [22].

In our study, a total of 411 students were selected randomly from three different colleges (CPHI, CAP, and CALSS) with a response rate of 75%, using a simple random technique to ensure the randomization of our sample. Of the selected students, 51.9% were females, and 48.1% were males. The sex ratio in our sample is representative of the population.

We found that the prevalence of depression and anxiety among Qassim University students was 40.6% and 29.4%, respectively. A previous study conducted specifically on Qassim University’s medical students found the prevalence of depression to be 50.2% [23]. In Bangladesh, a study similar to ours conducted on university students during the COVID-19 pandemic using the PHQ-9 and GAD-7 demonstrated a higher prevalence of depression and anxiety (53.8% and 42.9% for depression and anxiety, respectively). However, it is important to clarify that the authors of the study conducted in Bangladesh did not report the prevalence of depression and anxiety in their research. Therefore, we had to analyze their data to identify the prevalence of depression and anxiety in their sample based on a cut-off score for the diagnosis of depression and anxiety similar to our cut-off score (10 points) [24].

Based on the PHQ-9 and GAD-7 scoring systems, the levels of depression and anxiety in our study were somewhat similar to those among high school students in the Qassim region, according to a study conducted in 2019 [25].

In our study, depression and anxiety were positively correlated with sex. Females had higher levels of depression and anxiety, and their prevalence among females was 49.5% and 37.1, respectively, while that among males was 29.7% and 20.0%, respectively. This gap in sex distribution is expected since females are more likely to be diagnosed with depression and anxiety than males [26,27]. In addition, we found that those with chronic diseases tended to have higher levels of depression, which is consistent with that reported in the literature. A study based on systematic reviews, meta-analyses, and evidence-based clinical practice guidelines published between 1995 and 2007 concluded an association between physical illness and mental conditions such as depression and anxiety [28]. We believe that the alignment of our study with the literature in demonstrating the same positive correlations of different factors with depression and anxiety enhances the credibility of our measurement of them.

Regarding the comparison between the three different colleges (CPHI, CAP, and CALSS), we found that the students of CALSS had significantly higher levels of depression and anxiety. CALSS is considered a social science college and in two published studies, social science students were found to have higher levels of depression and anxiety [29,30]. This could be explained by the usual relative uncertainty regarding job opportunities among graduates of social studies.

Currently, the impact of the COVID-19 pandemic on the mental health of the general population is well established. This study showed that university students are not immune to this issue. We recommend psychological intervention programs that aim to enhance students’ mental health, especially when in-person learning has returned; thus, the obstacle of students being distant from the university is eliminated. We recommend a national effort by the Ministry of Education to encourage coordination between universities in Saudi Arabia to formulate a conjoint strategy to enhance students' mental health.

This study had some limitations owing to the COVID-19 pandemic. First, we were compelled to distribute the questionnaires through e-mail and social media platforms, which made it difficult to avoid the misinterpretation of the questions by students. Second, we had missing data in the first part of the questionnaire (socio-demographic questions) because we were restrained by the fear of not achieving an acceptable response rate, which led us to not enquire about every socio-demographic question to reduce students’ withdrawal from the study owing to privacy concerns.

## Conclusions

The post-pandemic prevalence of depression and anxiety among Qassim University students was found to be high. Depression and anxiety were more common in females than in males. Students with moderate and advanced depression had more chronic diseases than those with no or mild depression. Moreover, no significant correlation was found between anxiety and age, marital status, nationality, income, and chronic diseases. Social science students, in particular, were found to have more depression and anxiety than others. Finally, our findings demonstrate the need for psychological intervention programs for students of Qassim University. Students’ visits to academic advisors should include mental health questions. Students must be encouraged to visit the psychiatrists’ clinics at the University’s medical city when needed.
